# Lupus myositis, a type I interferon driven necrotizing myopathy with regional heterogeneity

**DOI:** 10.21203/rs.3.rs-10072964/v1

**Published:** 2026-06-29

**Authors:** Arpita Beechar, Bonnie Bermas, Chengsong Zhu, Changhong Xing, Jaya Trivedi, Salman Bhai, Joan S. Reisch, Veena Rajaram, Elena Daoud, Chunyu Cai

**Affiliations:** 1. Department of Internal medicine, Division of Rheumatic Diseases, University of Texas Southwestern Medical Center, Dallas, Texas; 2. Department of Immunology, Microarray and Immune Phenotyping Core Facility, University of Texas Southwestern Medical Center, Dallas, TX USA; 3. Department of Pathology, University of Utah School of Medicine, Salt Lake City, Utah; 4. Department of Neurology, University of Texas Southwestern Medical Center, Dallas, Texas; 5. Peter O’Donnell Jr. School of Public Health, Department of Health Data Science and Biostatistics, University of Texas Southwestern Medical Center, Dallas, Texas; 6. Department of Pathology, University of Texas Southwestern Medical Center, Dallas, Texas

**Keywords:** Lupus myositis, systemic lupus erythematosus, necrotizing myopathy, MxA, interferon, inflammatory myopathies, immunohistochemistry

## Abstract

Lupus myositis (LM) is an underrecognized entity, whose pathological features significantly overlap with idiopathic inflammatory myopathies (IIMs). Currently, no standardized histopathological criteria or immunohistochemical (IHC) markers exist for the diagnosis of LM on muscle biopsy. We performed detailed histologic, immunohistochemical, ultrastructural, and spatial transcriptomic protein analyses on a stringent cohort of LM muscle biopsies, excluding patients with myositis-specific autoantibodies (MSA). Findings were compared with dermatomyositis (DM), immune-mediated necrotizing myopathy (IMNM), antisynthetase syndrome (ASyS), and non-diseased control muscle specimens. Among 1736 patients diagnosed with systemic lupus erythematosus (SLE) between 2010 and 2023, 32 muscle biopsies were identified in myositis patients without MSA. Twenty-two cases demonstrated a necrotizing myopathy with spatial and temporal heterogeneity, MxA-positive myofiber expression, and perivascular inflammation composed of mixed T and B cells. A “pan-fascicular necrotizing myopathy” pattern was a highly recognizable feature of LM, although minority of cases demonstrated a diffuse scattered or perifascicular damage pattern. An IHC profile of MxA+/MHC I+/MHC II+ reliably distinguished LM from other IIMs. Spatial transcriptomics analysis confirmed that type I interferon pathway or MHC I related mRNAs and proteins were the most deferentially expressed in myofibers, capillaries and inflammatory cells. Electron microscopy identified frequent endothelial tubuloreticular inclusions. The remaining 10 cases demonstrated nonspecific myositis on muscle biopsy and were clinically associated with significant higher frequencies of overlapping systemic rheumatologic features such as interstitial lung disease, Sicca syndrome, systemic sclerosis, and rheumatoid arthritis, suggesting overlap myositis rather than pure LM. In conclusion, the pathological hallmark of LM is a type I interferon driven necrotizing myopathy with perivascular mixed T and B cell inflammation. A combined IHC panel including MxA, MHC I and MHC II effectively differentiates LM from other inflammatory myopathies.

## Introduction

Systemic lupus erythematosus (SLE) is a prototypical autoimmune disease characterized by dysregulated type I interferon (IFN-I) signaling. Lupus myositis (LM) occurs in approximately 6–7% of patients with SLE^1, 2^, and remains poorly defined, particularly from a histopathological perspective. The European Neuromuscular Center (ENMC) has established comprehensive clinico-sero-morphologic diagnostic criteria and treatment guidelines for idiopathic inflammatory myopathies (IIMs) including immune-mediated necrotizing myopathy (IMNM)^3^, dermatomyositis (DM)^4^, antisynthetase syndrome associated myositis (ASyS)^5^, and sporadic inclusion body myositis(sIBM)^6^. However, no equivalent standards exist for inflammatory myopathies associated with systemic rheumatologic diseases such as LM. Consequently, muscle biopsies from these patients are often categorized as “overlap myositis”^7^ or “non-specific myositis”^8^, terms that lack mechanistic or diagnostic precision.

Prior cohort studies of LM^1, 2^ have reported heterogeneous pathological findings, frequently labeling cases as polymyositis, DM, or necrotizing myopathy based on historical frameworks that predate current ENMC classifications^9, 10^. Moreover, the coexistence of overlapping clinical or serological features between SLE and IIM in a subset of patients further confounds interpretation of muscle pathology. Given major differences in prognosis, malignancy risk, organ surveillance and treatment between SLE^11^ and IIM^12^ patients, accurate pathological distinction on muscle biopsy is clinically important.

While definitive etiologic classification of myositis based solely on histopathology is often challenging, immunohistochemical (IHC) analysis—including markers such as major histocompatibility complex class I (MHC I), class II (MHC II), and terminal complement complex (C5b-9)—can offer a cost-effective diagnostic approach to narrow down the differential diagnoses.^13^. SLE is strongly driven by IFN-I signaling^14^. We have previously shown that myofiber expression of myxovirus resistance protein A (MxA), a surrogate marker of IFN-I activation, is common in LM but rare in non-dermatomyositis IIMs^15^.

In the present study, we performed a comprehensive histological, immunohistochemical, ultrastructural, and spatial transcriptomic analysis of a rigorously defined cohort of LM muscle biopsies, excluding cases with myositis-specific autoantibodies or alternative diagnoses. We compared these findings with DM, IMNM, and ASyS to define reproducible pathological features that distinguish LM, and propose a practical diagnostic framework for LM on muscle biopsy.

## Methods:

### Case selection:

The *LM cohort:* from a total of 1736 patients diagnosed with SLE between 2010 and 2023, 51 muscle biopsies from 50 patients were identified. Clinical information was reviewed by two rheumatologists (BB, AB); and all muscle biopsy slides and electron microscopy images were reviewed by two neuropathologists (CC, CX). Eighteen cases were excluded after review, those included 4 patients with co-existing MSA (two with Mi-2, one each with PL-12 and SRP autoantibodies), 3 patients who did not fulfil the 2019 European League Against Rheumatism/American College of Rheumatology Classification Criteria (EULAR/ACR) SLE diagnosing criteria^16^, and 11 muscle biopsy with non-myositis diagnoses (5 hydroxychloroquine myopathy, 3 vasculitis, 1 critical illness myopathy with thick filament loss, 1 normal muscle, and 1 with inadequate tissue from needle biopsy). The remaining 32 biopsies were included in this study.

Control myositis cohorts: The *DM cohort (n=15)* included muscle biopsies from a previously published cohort^17^ of juvenile DM patients with positive DM specific serum autoantibodies (10 NXP2, 4 Mi2, 1 TIF1γ). *The IMNM cohort (n=22)* included muscle biopsies with pathology features meeting the 2018 ENMC international workshop diagnostic criteria of IMNM^3^. Among those, 16 patients had positive anti-HMG-CoA reductase (HMGCR) autoantibody, 5 had positive anti-signal recognition particle (SRP) antibody, and one was seronegative. *The ASyS cohort (n=12)* included muscle biopsies meeting the 2024 ENMC clinico-sero-morphological criteria for ASyS^5^, in patients with ASyS specific autoantibodies (7 Jo-1, 2 PL7, 1 PL12 and 2 EJ).

This study was approved by the UT Southwestern Medical Center Human Research Committee on an excess tissue waived consent protocol. The study adhered to the principles outlined in the 1964 Declaration of Helsinki, and patient and control data were anonymized following the guidelines of the local institutional ethics review board.

### Muscle biopsy evaluation

Routine enzyme histochemical stains of muscle were performed on whole cryostat sections from the CLIA approved neuropathology clinical laboratory, ad included H&E, Gomori trichrome, nicotinamide adenine dinucleotide-tetrazolium reductase (NADH-TR), myosin ATPases at pH 9.4, pH 4.6 and pH 4.3, acid phosphatase, alkaline phosphatase, esterase, succinate dehydrogenase (SDH), cytochrome C oxidase (COX). IHCs performed on whole tissue section from cryostat sections included MxA (Sigma Aldrich, Anti-MxA, clone M143, MABF938, Burlington, MA), MHCI (Thermo Fisher Scientific, MA5–11723, HLA-ABC monoclonal antibody, clone W6/32, Waltham, MA), MHCII (Thermo Fisher Scientific, MA1–25914, HLA-DR/DP/DQ monoclonal antibody, clone CR3/43, Waltham, MA), C5b-9 (Dako/Agilent, terminal complement complex, clone aE11, Santa Clara, CA). IHCs performed on formalin fixed, paraffin embedded muscle sections included CD3 (Dako/Agilent, clone F7.2.38, Santa Clara, California, USA.), CD20 (Thermo Fisher Scientific, MA5–41781, Waltham, MA) and CD68 (Thermo Fisher Scientific, MA5–13324, KP1 clone, Waltham, MA) Scoring of muscle pathology in myofiber, inflammation, connective tissue and vascular domains and MHCI/MHCII/MxA IHCs were detailed in supplementary method.

Electron microscopy (EM) was performed on majority of myosiitis biopsies for evaluation of endothelial tubuloreticular inclusion (TRI). For each case more than 20 capillaries were examined under high magnification on EM. Presence of endothelial TRI was scored as 0 (none), 1 (found only in one or two capillaries), 2 (found in more than two capillaries).

Statistical analyses were performed using MedCalc Version 20.009. Fisher’s exact test was used to compare groups for categorical measures and the Mann-Whitney U test was used for numerical measures. Significance was selected as *p* ≤ 0.05. No adjustment was made for multiple comparisons.

#### GeoMx Whole Transcriptome Atlas (WTA) and Immuno-Oncology Proteome Atlas (IPA)

The GeoMx WTA plus IPA proteogenomic assay allows for the profiling of human whole transcriptomes combined with over 570 photocleavable, oligo-tagged antibody targets that can be analyzed on the same slide. The data for the GeoMx IPA were measurements of protein abundance of 570 proteins, five housekeeping proteins (Histone H3, GAPDH, RPS6, Calreticulin and TOMM20) and five background control (Rat IgG2a, Mouse IgG2b, Hmr IgG, Rabbit IgG and Mouse IgG1) probes. The staining protocol for the WTA/IPA experiment was carried out as per the manufacturer’s guidelines. To perform the GeoMx^®^ assays, 5 μm FFPE sections were mounted on charged slides, baked, and prepared on the Leica Biosystem, following the manufacturer’s automated slide preparation user manual. After hybridization with protein probes that are conjugated to barcoded oligonucleotide tags with an ultraviolet (UV) photocleavable linker and staining with fluorescent morphology markers consisting of desmin (myofiber), CD34 (endothelial cells), CD45 (immune cells), SYTO13 (nuclear), the slides were loaded onto a GeoMx^®^ digital spatial profiler (DSP, NanoString Technologies) and scanned. The stained tissue sections were subsequently processed using the NanoString Technologies GeoMxDigital SpatialProfiler (DSP) to collect oligonucleotide tags attached to conjugated antibodies within user-defined regions of interest (ROIs). After labeling, the slides were imaged and interested cell were selected. Subsequently, the selected cells were exposed to UV light, and the barcoded oligos were released, aspirated, and dispensed into a collection plate for library construction for next-generation sequencing (NGS).

#### NGS library preparation and sequencing

GeoMx libraries were prepared following the manufacturer’s instructions. After the mRNA collection was complete, aspirates in the collection plate were dried at 65°C for 1 hour in a thermal cycler with an open lid and resuspended in 10 μL of nuclease-free water. 4 μL rehydrate aspirates were mixed with 2 μL of 5×PCR Master Mix and 4 μL of Procode primers. PCR amplification was then performed with 18 cycles. The indexed libraries were pooled equally and purified twice with 1.2×AMPure XP beads (Beckman Coulter, Brea, CA). The final libraries were evaluated and quantified using Agilent’s High Sensitivity DNA Kit and Invitrogen’s Qubit dsDNA HS assay, respectively. Total sequencing reads per DSP collection plate were calculated using the NanoString DSP Worksheet. The libraries were sequenced using 38 bp paired-end sequencing (PE 38) on an Illumina NovaSeq 6000 system with a 100-cycle S1 kit (v1.5). The GeoMx NGS Pipeline (v3.2.0.46) processes RNA-sequencing files (FASTQ files) from Illumina sequencers according to parameters defined in the Configuration File (which is generated from the GeoMx DSP run). The Pipeline processes information from these files and outputs DCC files were uploaded to the GeoMx DSP system for data analysis.

#### Data processing and analysis

Data obtained from the NanoString GeoMx DSP platform were analyzed using a dedicated software (GeoMx Analysis Suite 2.3). The quality control analysis showed that all ROIs had raw read counts above 1 million and good alignment rate and sequencing saturation. The local outliers were negative control probes and were removed from the ROIs. ROIs with deduplicated probe counts Q3 (upper quartile) < 2 were excluded. Furthermore, ROIs were removed if < 1% of their genes had read counts higher than the limit of quantification (LOQ). LOQ was defined as geomean (NegProbe) × geoSD (NegProbe)^2^ for each ROI. Target filtering was applied to retain gene targets with read counts above LOQ in at least 10% of ROIs. This resulted in 12,499 genes (out of 18 000+). Q3 normalization was then applied on the filtered ROIs and protein targets.

Protein expression across groups was analyzed by limma package; differentially expressed proteins (DEPs) were defined as fold-change > 1.5 or < −1.5, and Benjamini-Hochberg-adjusted p < 0.05. Analyses were conducted using R software (version 3.2.2; R Foundation for Statistical Computing, Vienna, Austria). The heatmap was generated by R pheatmap package.

## Results

### Clinical and serological characteristics of patients with lupus myositis

Among 32 patients included in this study, 91% were females with the mean age of 31 (range: 7–55) at time of muscle biopsy. Myositis was the initial presentation of SLE in 31% of the patients. In the remaining patients with previously diagnosed SLE, the mean time between the initial SLE diagnosis and the muscle biopsy was 6 years (range 0–10 years). The main presenting symptoms of myositis were proximal weakness (95%), myalgia (85%) and creatine kinase (CK) elevation (90%). The mean peak CK was 3,969 IU/L (range 92–26,000). Serology was significant for positive antinuclear antibody (ANA,90%), anti-Smith antibody (73%), anti-double strand DNA (76%), anti-ribonucleoprotein antibody (63%) and hypocomplementemia (66%). Most patients had other systemic manifestations, including 69% patients with rashes, alopecia or other skin/mucosal findings, 78% with arthritis, 75% with leucopenia and/or anemia, 47% with serositis or myocarditis, and 25% with biopsy proven lupus nephritis.

### Pathologic features of lupus myositis

Majority (n=22) of the LM demonstrated a necrotizing myopathy accompanied by perimysial/perivascular mononuclear inflammation. By temporal progression the muscle pathology can be divided into 3 phases. Early phase was characterized by *vacuolar basophilic changes* in affected myofibers, often with increased internalized nuclei (Fig 1A, inset). Frankly necrotic fibers, highlighted by C5b-9 (Fig 1B, arrows), were sparce at this phase, corresponding with only mildly elevated CK levels (mean:503, range 292 to 713 U/L). C5b-9 may demonstrate patchy strong capillary reactivity (Figure 1B, upper right). Regenerating fibers and connective tissue damage were typically rare or absent at this phase (Fig 1C, alkaline phosphatase). IFN-I surrogate marker MxA was strongly expressed in myofibers with mosaic staining intensity (Fig 1D). The *middle - necrotic phase* was characterized by abundant necrotic fibers. Patients’ CK were typically very high (mean: 5853 U/L, range: 1319 – 12755 U/L). Some of the necrotic fibers were centrally necrotic, peripheral regenerating (CNPR) fibers (Fig 1E arrow), previously reported as a specific feature for dermatomyositis^18^. C5b-9 highlighted the necrotic portion of the CNPR fibers (Fig1F arrow), while ALP strongly stained the basophilic regenerating portion of CNPR fibers (Fig 1G arrow). MxA myofiber expression was still present but markedly reduced compared to the early phase (Fig 1H). The *late - regenerating/fibrotic phase* was characterized by frequent small round fibers, interstitial fibrosis, and frequent internalized nuclei (Fig1 I). C5b-9 may demonstrate widespread sarcolemmal staining in viable myofibers (Fig 1J). ALP showed frequent regenerating fibers and strong, widespread endomysial and perimysial connective tissue reactivity (Fig 1K). MxA was largely absent in myofibers at this stage except for isolated weakly positive myofibers. (Fig1M, arrow)

Using MxA positive myofibers as an indicator for active disease, we identified different spatial distribution patterns. The most common injury pattern was a unique *pan-fascicular necrotizing myopathy (PanF NM*, 13/22, 54%)^15^, where injured fibers were concentrated in some fascicles more than others, and the injured fibers were distributed throughout the involved fascicles without preference to perivascular regions or vascular territories (Fig2A–C, Supplementary Fig1). A *perifascicular necrotizing myopathy* *(PeriF NM*, 5/24, 21%) was the second most common pattern (Fig2 D), with MxA positive fibers preferentially located to the perifascicular regions. A *diffuse scattered necrotizing myopathy* *(Diffuse NM* Fig2 G–I) was seen in a minority (4/24,17%) of cases, where MxA positive fibers were diffusely and randomly distributed throughout the entire muscle biopsy without significant inter- or intra-fascicular variation. MHCI was diffusely and strongly upregulated in the sarcolemma of all myofibers in majority of cases regardless of necrotic fiber distribution (Figure 2 B, E, H). MHCII expression was weakly positive (Fig 2 C, F, I), intermediate between dermatomyositis (negative) and inclusion body myositis (strongly positive).

Perivascular inflammatory aggregates were present in 19/24 (79%) of cases. Those included 77% cases with PanF, NM, 60% of periF NM and 100% of diffuse NM. The inflammatory aggregates were composed of mixed B cells, T cell, histiocytes, and occasionally plasma cells. Three cases had large lymphoid aggregates with germinal centers.

The remaining 10 cases showed nonspecific myositis that did not cleanly fall into the above necrotizing myopathy patterns. Among those, two muscle biopsies demonstrated a polymyositis (PM) pattern characterized by predominance of endomysial lymphohistiocytic inflammation (Fig2, J–L), fulfilling the 205^th^ ENMC International Workshop definition of PM^8^. The remaining 8 cases showed focal perivascular inflammation (n=3), patchy MHCI upregulation in myofibers (n=2), or rare isolated necrotic/regenerating fibers (n=3).

#### EM features of active lupus myositis.

EM was performed for all cases. Endothelial TRI was found in 17/22 (77%) cases of the necrotizing cases, regardless of necrotic fiber distribution pattern, but only 1/10 (10%) of the nonspecific myositis cases. In majority of the necrotizing cases, the TRI were frequent and large/well formed (Fig 3A, arrow). There was a moderate positive correlation between presence of TRI and myofiber MxA expression (Pearson correlation coefficient r =0.48). Capillary basement membrane (BM) was often thickened, while BM reduplication was not a prominent feature (Fig 3A). Nonspecific myopathic findings were variably present and included Z band streaming (Fig3B), sarcomeric disarray with cytoplasmic bodies (Fig 3C), clear vacuoles (Fig 3D), degenerating fiber with increased autophagic bodies (Fig 3E), to frankly necrotic fiber (Fig3F, single asterixis) and regenerating fiber (Fig3F, double asterixis). Intranuclear inclusions were not identified in any case.

#### Clinical pathological correlation:

Patients with necrotizing LM had significantly higher rates of characteristic SLE clinical features (Table 2), including lupus nephritis, low C3 and/or C4, and a trend towards increased frequency of antiphospholipid syndrome (obstetric complications, cerebral vascular accidents). On the other hand, patients with nonspecific myositis had significantly higher frequencies of overlap features of other systemic rheumatology diseases including ILD, sicca syndrome, positive rheumatoid factor (RF), and a trend towards increased scleroderma features. The two patients with PM features on muscle biopsy (Figure 2J–L) had very high titer RF and inflammatory arthritis that responded poorly to hydroxychloroquine and methotrexate.

#### Comparative study between LM vs DM, IMNM and ASyS, respectively.

To better differentiate LM from other myositis on muscle biopsies, we performed detailed pathology analysis on the 22 cases of LM vs 15 cases of DM, 22 cases of IMNM, and 12 cases of ASyS. Pathological features from four domains: inflammation, myofiber, connective tissue and vasculature were scored for each case. The scoring criteria were detailed in supplementary methods. The mean scores for each variable were listed in table 2. Mann-Whitney tests were performed to compare LM to each of the other myositis groups, respectively.

##### LM vs DM

LM and DM differed significantly over the myofiber and connective tissue domains and IHC (Table 2). Overall, a PanF distribution of myofiber pathology was the most useful feature to distinguish LM from DM. Secondly, DM was dominated by perifascicular atrophy, while LM was dominated by myofiber necrosis and regeneration. One notable exception was the DM-Mi2 variant, which had more prominent perifascicular myofiber necrosis/regeneration than other DM variants^17, 18^. Distinction between DM-Mi2 and a minority of LM with a perifascicular necrotizing pattern can be very difficult on pathology alone. Immunohistochemically, MHCII was usually weak or negative in DM but moderate in LM. MxA and MHCI were positive in both conditions and less differential.

##### LM vs IMNM

LM and IMNM were significantly different from all domains. LM had more prominent lymphocytic and macrophagic inflammation, diffuse or patchy MHCI/MHCII upregulation, and MxA myofiber positivity. PanF injury pattern was never observed in IMNM cases. Vascular damages, including both perimysial arteries and capillaries abnormalities, were substantially higher in LM than IMNM. Perimysial and endomysial fibrosis and ALK phosphatase activity were also common in LM, but rare in IMNM. Immunohistochemically, IMNM was always negative for MxA and nearly always negative for MHCII. MHCI, when present, was only in scattered regenerating and myophagocytic fibers and rarely diffuse or patchy.

##### LM vs ASyS

In ASyS, necrotic fibers were more restricted to perifascicular regions; while in LM, regions of PeriF necrosis often co-existed with regions of PanF necrosis in the same biopsy. ASyS cases also had the most prominent perimysial connective tissue damage reflected in the strongest ALK phosphatase connective tissue reactivity and the highest MHCII myofiber expression among all myositis analyzed, which were significantly stronger than LM. MxA was usually negative in ASyS, although equivocal staining in necrotic fibers was sometimes difficult to differentiate from true positive fibers. On EM, endothelial TRI could be present in minority of cases of ASyS but much less frequent than DM. Intranuclear actin aggregation^19^ was a hallmark for ASyS and never observed in LM, DM or IMNM.

#### Spatial transcriptome study:

**Figure 4:**
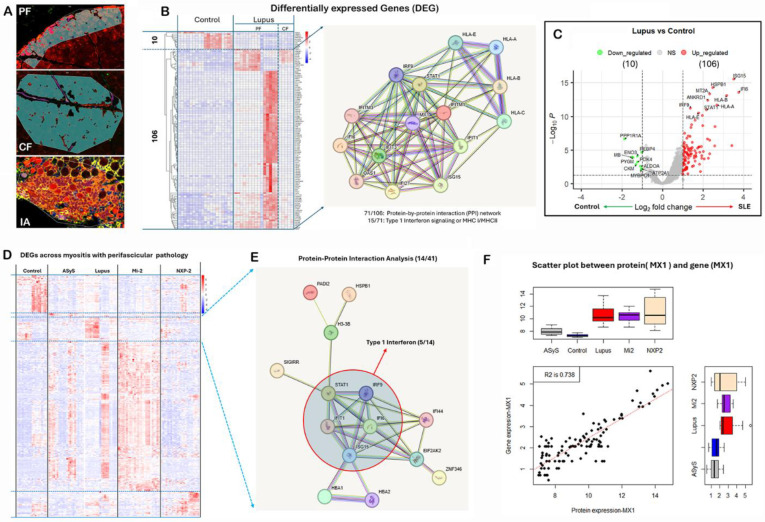
Spatial transcriptome analysis on lupus myositis. A: Immunofluorescence imaging of muscle. The regions of interest were perifascicular (PF), centro-fascicular (CF), and inflammatory aggregates (IA). B: The most deferentially expressed genes (DEGs) and Protein-Protein Interaction (PPI) analysis between LM and normal control. C: Volcano plot between LM and control muscle. D: DEGs comparison across myositides and E: PPI analysis highlights IFN1 signature in LM. F:Gene and protein correlation plot for INF-I signature MX1.

LM, DM-Mi2 and ASYS can all demonstrate a perifascicular necrotizing myopathy pattern, while DM-NXP2 usually demonstrates a perifascicular atrophy pattern. To investigate difference in their underlining disease mechanisms, we performed spatial transcriptome analysis by Nanostring GeoMx Digital Spatial Profiler (DSP), compared mRNA and protein expression profiles on muscle tissue from two of each LM, DM-NXP, DM-Mi2, ASYS-Jo1 and age matched non-disease control muscle biopsies (10 case total). Desmin, CD34 and CD45 immunofluorescence markers were used to identify the myofiber, vessels and inflammation domains, respectively. Regions of interest (ROI) were drawn over perifascicular (PF) regions where myofiber pathology were most prominent versus centro-fascicular (CF) regions where myofiber pathology were minimal in each myositis conditions, and inflammatory aggregates (IA) (Fig 4A).

Spatial transcriptomic analysis of 14,000 mRNAs revealed a robust and disease-specific immune activation signature in LM. Differential mRNA expression analysis identified 106 upregulated differentially expressed genes (DEGs) in LM compared with normal controls (Fig 4B). Notably, the most significantly upregulated genes were associated with IFN-I pathway and antigen processing and presentation pathways (MHC I & II), highlighting the central role of IFN-driven inflammation (Fig 4C). Comparative analyses across myositis subtypes further demonstrated a distinct LM transcriptional profile of 41 DEGs (Fig 4D). Analysis by STRING 10 software (https://string-db.org/) found 14 DEGs linked by Protein-Protein Interaction (PPI) network, of which half were from IFN-I pathway (Fig4 E). Integration mRNA with proteomic data demonstrated strong concordance between gene and protein expression, particularly for IFN-stimulated genes (IFIT1, ISG15, STAT1, MX1) and components of antigen presentation machinery (MHCI/MHCII), supporting the functional relevance of these pathways (Fig4 F and supplementary Fig 2).

Protein analysis of the most increased differentially expressed proteins (DEPs) similarly demonstrated IFN-I signature proteins (ISG15, MX1, IFIT1) highly expressed in LM, DM-Mi2, and DM-NXP2, but low in ASyS and normal controls (Supplementary Fig 3), while MHC II signature proteins (HLA-DP, HLA-DR) were most highly expressed in ASyS. When comparing myositis specific DEPs, necrosis/apoptosis markers (caspase3/AIF/ASC were strongly overexpressed in DM-Mi2, LM and ASYS, while hypoxia markers CA3/CA9 were strongly overexpressed in DM-NXP2. Wnt pathway markers beta-catenin/SFRP4 were overexpressed in DM-NXP2 and DM-Mi2 variants, Shh pathway marker PTCH2 was overexpressed in LM, and extracellular matrix remodeling proteins NCAM1/fibronectin/MMP7,8,9 were overexpressed in ASyS. Notably, MAO-A had higher protein expression in LM than any other myositis, predominantly in the CF region myofibers, likely reflecting a high responsiveness to cortical steroid in these fibers. (Supplementary figure 4).

## Discussion

This study identifies lupus myositis as a distinct pathological entity characterized by a IFN-I–driven necrotizing myopathy with marked spatial and temporal heterogeneity. The most diagnostically useful feature is a PanF necrotizing myopathy accompanied by mixed perivascular inflammation and evidence of IFN-I pathway activation.

A combined immunohistochemical profile of MxA, MHC I, and MHC II reliably distinguished LM from major IIM subtypes. Overall, a MxA+/MHCI+/MHCII+ IHC profile supported the diagnosis of LM, a MxA+/MHCI+/MHCII- IHC profile supported the diagnosis of DM; a MxA-/MHCI+/MHCII+ IHC profile supported the diagnosis of ASyS; and a MxA-/MHCI- (mosaic)/MHCII- profile supported the diagnosis of IMNM. MHCII was strong in ASyS, medium in LM, weak to negative in DM and negative in IMNM.

A subset of lupus patients had a muscle biopsy that demonstrated nonspecific inflammatory myopathy findings. Most of those patients demonstrated overlapping clinical features of other systemic rheumatologic diseases. For example, two muscle biopsies demonstrated endomysial dominant PM type inflammation. There was no increase in ragged red fibers, COX deficient fibers, or red rimmed vacuoles to suggest inclusion body myositis or polymyositis with mitochondrial pathology (PM-mito)^20^. Both biopsies were from patients with high titer RF autoantibodies and erosive inflammatory arthritis in addition to lupus clinical features. This same histological pattern has also been observed at our institution in muscle biopsies from “pure” rheumatoid arthritis patients without lupus features. Other patient with nonspecific myositis findings had significantly higher rates of overlapping clinical features such as ILD, Raynaud phenomenon, Sicca symptoms, and scleroderma skin changes. We speculate that their muscle pathology likely represented overlap myositis rather than pure lupus myositis.

Spatial transcriptomic analysis further reinforced robust IFN-I, MHCI and MHCII related genes and protein expressions in LM muscle biopsiies. We also demonstrated high expression of Monoamine oxidase A (MAO-A) in LM relative to other IIMs. MAO-A is a major target gene for glucocorticoids in human skeletal muscle cells^21^. We found MAO-A expression was higher in CF regions (more normal) than PF regions (more damaged), suggesting a protective effect on muscle fibers in LM muscle biopsy. Whether LM is more responsive to glucocorticoids treatment than other IIMs warrants further investigation.

Our findings expanded prior observations by integrating morphology, immunophenotype, ultrastructure, and spatial transcriptomics. We also demonstrated that IFN-I signaling was most prominent in early disease stages and diminished with disease progression, providing a framework for interpreting variable MxA expression in diagnostic biopsies.

Limitations: This was a retrospective study from a single institution and subject to selection bias. Clinical, serological and pathological data were incomplete in some cases due to the nature of a muscle biopsy reference center. The spatial transcriptome study was limited by small case number (2 per myositis types) and subject to individual case variability. Nonetheless, this study establishes reproducible pathological criteria for LM and provides practical diagnostic guidance for routine neuropathology practice.

In conclusion, the hallmark of LM pathology is a IFN-I pathway driven necrotizing myopathy with perivascular mixed T and B cell inflammation. A panfascicular necrotizing myopathy pattern was highly recognizable and a useful diagnostic feature on muscle biopsy. An IHC panel including MxA/MHCI/MHCII effectively differentiates LM from other inflammatory myopathies.

## Supplementary Material

Supplementary Files

This is a list of supplementary files associated with this preprint. Click to download.

- supplementarydata.docx

## Figures and Tables

**Figure 1:** Temporal progression of muscle pathology in lupus myositis. **(A-D): early vacuolar basophilic phase.** (A) H&E, inset showed vacuoles in higher magnification. (B) C5b-9 (C) ALP. Arrows in A, B, C pointed to frankly necrotic fibers. (D) MxA. **E-H: Middle necrotic phase.** (E) H&E, (F) C5b-9, (G) ALP. Arrow in E, F, G pointed to a cluster of centrally necrotic, peripheral regenerating (CNPR) fibers. (H) MxA. (**I-M): Regenerating-fibrotic phase.** I) H&E, (J) C5b-9, (K) ALP, (M) MxA. (H&E, C5b-9, ALP were images from the same region on consecutive cryostat sections of fresh frozen tissue. MxA were images from formalin fixed, paraffin embedded tissue of corresponding cases.)

**Figure 2:** Spatial heterogeneity of myofiber involvement in lupus myositis subtypes. (A-C): Panfascicular necrotizing myopathy (PanNM) pattern. (D-F) Perifascicular necrotizing myopathy (PeriNM) pattern. (G-I): Diffuse necrotizing myopathy (Diffuse NM) pattern. (J-L) Polymyositis (PM) pattern. Necrotic and regenerating fibers were enriched in MxA (A, D, G, J) positive regions. MHC1 (B, E, H, K) was diffuse and strong in most cases regardless of subtypes. MHC2 (C, F, I, L) was weak except for the PM pattern.

**Figure 3:** EM features of active lupus myositis. (A) Endothelial tubuloreticular inclusion (arrow). (B) Z band streaming. (C) Complete sarcomeric disarray and cytoplasmic bodies. (D) Clear vacuoles (E) Degenerating fiber with increased autophagic bodies. (F) Necrotic fiber (single asterixis) and regenerating fiber (double asterixis).

**Table 1:** Clinical and laboratory features of patients diagnosed with systemic lupus erythematosus and necrotizing vs nonspecific inflammatory myositis

	Necrotizing (n=22)	Nonspecific (n=10)	*p*-value
Median age （range）	32 years (18–70)	24 (5–55)	0.272*
Sex Ratio (female/male)	19/3	10/0	0.530
CK Mean (Range)	4,857 (292–26,000)	1,838 (92–7,555)	0.226*
Clinical features			
Mucocutaneous	15 (68%)	7 (70%)	1.00
Arthritis	16 (73%)	9 (90%)	0.39
Raynaud’s	6 (29%)	6 (60%)	0.12
Sicca	3 (14%)	6 (60%)	**0.013**
Hematologic	15 (68%)	9 (90%)	0.38
Serositis	11 (50%)	4 (40%)	0.71
Lupus Nephritis	8 (36%)	0 (0%)	**0.035**
ILD	1 (5%)	4 (40%)	**0.024**
Overlap Systemic Sclerosis	1 (5%)	3 (30%)	0.079
Obstetric Complications/CVA	7 (32%)	0 (0%)	0.069
Laboratory features			
ANA	18 (95%)	8 (80%)	0.26
Anti-Smith	16 (80%)	6 (60%)	0.38
Anti-RNP	13 (65%)	6 (60%)	1.0
Anti-dsDNA	14 (74%)	8 (80%)	1.0
SSA/SSB	9 (45%)	6 (60%)	0.7
Low C3 or C4	15 (79%)	4 (40%)	**0.05**
RF	1 (5%)	5 (50%)	**0.006**
CCP	2 (10%)	0 (0%)	0.54
ANCA	2 (10%)	3 (30%)	0.3

Fisher two sided exact test was used to calculate p values for binary variables.

* Mann-Whitney tests were used to calculate p values for continuous variables.

**Table 2:** Pairwise comparison of pathologic feature between LM vs. DM, IMNM and ASYS.

Pathology Scores Median (95% CI for median)	LM (n=22)	DM (n=15)	IMNM (n=22)	ASys (n=12)	*P* value* LM vs DM	*P* value* LM vs IMNM	*P* value* LM vs ASyS
**Inflammation Domain**							
Lymphocytic inflammation (0–2)	1.0 (1.0–1.1)	2 (0–2.0)	0 (0–0)	2 (1.2–2.0)	0.50	**<0.0001**	**0.03**
Macrophagic inflammation (0–2)	2.0 (1.0–2.0)	1.0 (1.0–1.7)	0.5 (0–1.0)	1.0 (1.0–2.0)	**0.01**	**0.0001**	0.14
**Vascular Domain**							
Arterial abnormality (0–1)	1.0 (1.0–1.0)	1.0 (1.0–1.0)	0 (0–0)	1.0 (1.0–1.0)	0.83	**<0.0001**	0.91
Capillary TRI (0–2)	2.0 (1.0–2.0)	2.0 (1.9–2.0)	0 (0–0)	0 (0–0.83)	0.22	**0.0005**	**0.001**
**Myofiber Domain**							
Myofiber atrophy							
Pan fascicular (0–2)	0 (0–1.0)	0 (0–0)	0 (0–0)	0 (0–0)	0.06	**0.001**	**0.04**
Perifascicular (0–2)	0 (0–0)	2.0 (1.3–2.0)	0 (0–0)	0.5 (0–1.0)	**<0.0001**	0.08	0.14
Diffuse scattered (0–1)	0 (0–1.0)	0 (0–1.0)	1.0 (0.95–1.0)	1.0 (0–1.0)	**0.005**	**0.04**	0.26
Myofiber damage							
Pan fascicular (0–2)	1.0 (0–2.0)	0 (0–0.7)	0 (0–0)	0 (0–0)	**0.02**	**0.0006**	**0.02**
Perifascicular (0–2)	0 (0–1.0)	2.0 (1.0–2.0)	0 (0–0)	1.5 (1.0–2.0)	**0.0004**	**0.005**	**0.01**
Diffuse scattered (0–1)	0 (0–1.0)	0 (0–0)	1.0 (1.0–1.0)	1.0 (0–1.0)	0.07	**0.0001**	0.57
ALK phos positive regen. fiber (0–2)	1.0 (1.0–1.0)	1.0 (0.3–1.0)	1.0 (1.0–2.0)	1.5 (1.0–2.0)	0.13	0.40	0.42
Internal nuclei (0–1)	1.0 (1.0–1.0)	0 (0–1.0)	1.0 (0–1.0)	1.0 (0–1.0)	**0.004**	**0.04**	0.07
**Connective Tissue Domain**							
ALK phos perimysial reactivity (0–2)	1.5 (1.0–2.0)	0 (0–1.0)	0 (0–1.0)	2 (2.0–2.0)	**0.03**	**0.03**	**0.03**
Perimysial fibrosis (0–1)	1.0 (1.0–1.0)	1.0 (1.0–1.0)	0 (0–0)	1.0 (0–1.0)	0.34	**<0.0001**	**0.03**
Endomysial fibrosis (0–1)	0.5 (0–1.0)	0 (0–1.0)	0 (0–0)	0 (0–0)	0.55	**0.03**	0.059
**IHC**							
MHC1	2 (1.0–2.0)	2 (2.0–2.0)	0 (0–1.0)	2 (2.0–2.0)	**0.046**	**<0.0001**	0.09
MHC2	1.20	0.18	0.09	2.0	**0.0004**	**0.0001**	**0.003**
MxA	2 (1.0–2.0)	2 (2.0–2.0)	0 (0–0)	0 (0–1.0)	0.339	**<0.0001**	**0.0004**
C5b-9 fiber	1.5 (1.0–2.0)	1.0 (1.0–2.0)	2 (1.0–2.0)	2 (1.0–2.0)	0.48	0.89	0.84
C5b-9 capillary	0 (0–1.1)	1.0 (1.0–1.5)	0 (0–0)	0 (0–0)	0.11	0.067	0.11

* Mann-Whitney tests were performed on all categorical variables.

## Data Availability

The GeoMx data were deposited into the GEO database under accession number GSE331197(WTA) and GSE330891(IPA), *following the URL:* https://www.ncbi.nlm.nih.gov/geo/query/acc.cgi?acc=GSE330891 *and* https://www.ncbi.nlm.nih.gov/geo/query/acc.cgi?acc=GSE331197
